# Series arc fault detection based on continuous wavelet transform and DRSN-CW with limited source data

**DOI:** 10.1038/s41598-022-17235-7

**Published:** 2022-07-27

**Authors:** Congqiang Hu, Na Qu, Shuai Zhang

**Affiliations:** School of Safety Engineering, Shenyang Aerospace University, Shenyang, 110136 China

**Keywords:** Engineering, Mathematics and computing

## Abstract

When a series arc fault occurs in an indoor power distribution system, the temperature of arc combustion can be as high as thousands of degrees, which can lead to an electrical fire. Deep learning has developed rapidly in recent years and is widely used in fault diagnosis. The problem is that the sourced data is challenging to obtain, and few public data sources affect the application of deep learning models in arc fault diagnosis. In order to solve this problem, an arc fault detection method based on continuous wavelet transform and deep residual shrinkage network with the channel-wise threshold (DRSN-CW) is proposed. First, the grayscale images of source data features are obtained by continuous wavelet transform. Then, the feature images are data enhanced to construct the dataset. Finally, the DRSN-CW model is constructed and used to detect arc fault. The results show that the highest accuracy of arc fault detection is 98.92%, and the average accuracy is 97.72%. This method has excellent performance, which provides a new idea for arc fault detection.

## Introduction

Electrical fire generally refers to spontaneous combustion or combustion of other combustibles caused by high temperature and arc caused by faults of electrical circuits and equipment. If the insulation layer of electrical wires or appliances is aged or damaged and the insulation ability will decline, or the voltage reaches a specific value, an arc fault may occur. Moreover, when a series arc fault occurs, the current value is often less than the threshold of the circuit breaker. According to the analysis of relevant data, the series arc fault of the power distribution system has become one of the leading causes of electrical fires^1^. Therefore, detection of the arc fault is important to ensure electrical safety. At present, the methods of arc fault detection are divided mainly into three categories. First, sensor technology is used to detect arc light, arc sound, temperature, electromagnetic radiation, and other arc indicators to judge the occurrence of an arc fault. This method has high requirements for the environment and installation position and has limitations in practical use. It is generally used to detect arc fault in specific switchgear^2–5^. The second is the simulation research of the arc mathematical model, which mainly includes the Cassie, Mayr, Schavemaker, and Habedank models^6–9^. It uses computer simulation technology instead of complex arc experiments. However, due to the limitations of the parameters and the application conditions, the mathematical model cannot simulate arc faults completely and accurately^10^. The third is based on the analysis of the current signal, which is the leading research direction on arc fault at present. The analysis focuses mainly on the time–frequency features of the current signal^11–13^.

In recent years, with the rise of artificial intelligence, intelligent detection algorithms have been widely used in the field of fault diagnosis. It provides new ideas for the detection of series arc faults. In particular, deep learning detection algorithms such as CNN^14–17^, AlexNet^18,19^, and ResNet^20^ have been combined with arc fault features to achieve excellent results in the field of arc fault detection. It has become an important research direction for the current arc fault detection. However, the above methods ignore the fact that in the actual electrical environment, the collected arc fault sample data is limited and the noise data has a huge impact on the detection results. It cannot meet the requirements of the detection algorithm with deep learning. Therefore, in this paper, a detection method of series arc fault based on continuous wavelet transform and DRSN-CW is proposed. It can achieve the detection of arc faults with limited data samples and avoid the influence of noisy data on the detection results.

Toward this method, the main contributions of this paper are as follows:

1. A feature extraction method based on the continuous wavelet transform is proposed, which solves the problem that the features of the current signal are difficult to obtain.
2. A dataset enhancement method based on adding noise, rotation and mirroring is proposed for the problem of few fault arc data.
3. In this paper, an arc fault detection method based on DRSN-CW is proposed. Through the model-specific automatic soft thresholding, noise and redundant data are removed to avoid overfitting and accuracy degradation caused by noisy data.
4. Other detection models are explored and compared with the proposed DRSN-CW model. The superiority of the proposed method is demonstrated.

## Methods

### Data collection

**Figure 1:**
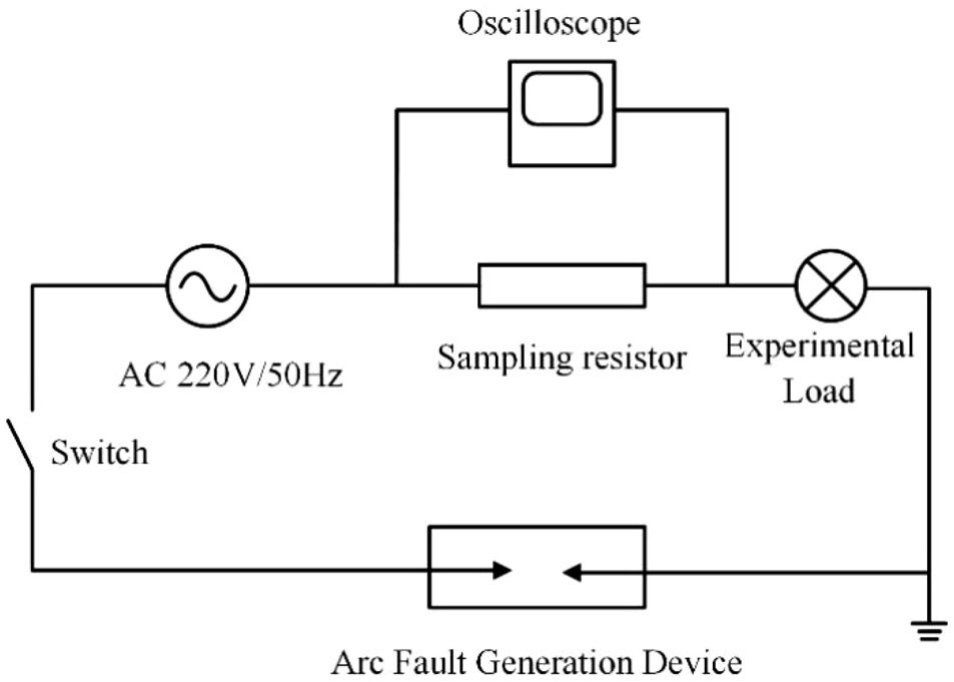
Series arc fault experimental principle.

**Figure 2:**
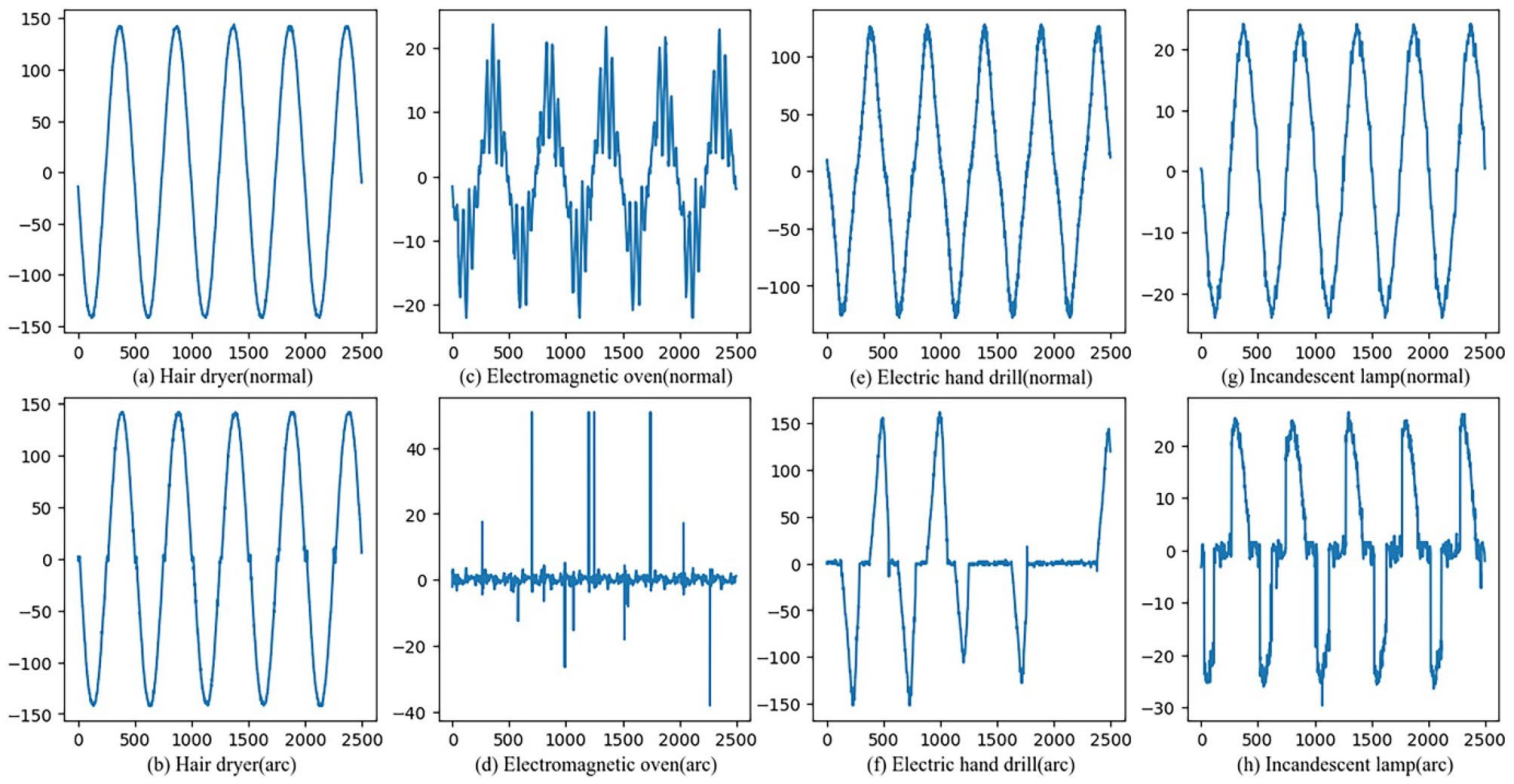
The time domain diagram of four typical load under normal and arc fault states.

According to the UL1699 international standard, a series arc fault experimental platform is constructed. Four typical appliances are selected as sampling loads, namely hair dryer, electromagnetic oven, electric hand drill, and incandescent lamp. The data of normal and arc faults are collected from different loads. The series arc fault experiment platform is mainly composed of arc fault generation device, experimental load, 220 V/50 Hz AC, sampling resistor, and an oscilloscope, as shown in Fig. 1. The sampling frequency is 25 kHz, which is set according to Nyquist's sampling theorem. The arc fault generation device is designed according to UL1699. It mainly consists of a fixed carbon rod electrode, moving metal electrode, and stepper motor. According to the standard, the fixed graphite electrode adopts a 6.4 mm diameter carbon rod, and the mobile metal electrode adopts a 10 mm diameter copper rod with sharpened contacts. These electrodes are controlled by a stepper motor with a speed of 0.2 mm/s, and move slowly until an arc fault occurs. The waveforms of the collected current signal from the four typical loads in normal and arc fault states are shown in Fig. 2.

### Feature extraction

In low-voltage distribution systems, series arc fault currents contain many non-smooth and random components, such as offsets, abrupt changes, and trends. Compared with the Fourier transform, the wavelet transform inherits and develops the idea of short-time Fourier localization and overcomes the shortcomings of the traditional Fourier transform. It can adapt to the requirements of time–frequency signal analysis, focus on arbitrary details of the signal, and facilitate the processing of abruptly changing signals such as arc faults.

Set $\psi (t) \in L^{2} (R)$, $L^{2} (R)$ denote the square-producible space of real numbers, the Fourier transform $\psi^{{\prime}} (\omega )$ satisfies the constraint that:

1 $$C_{\psi } = \int_{ - \infty }^{ + \infty } {|\psi^{\prime}(\omega )|^{2} \frac{{{\text{d}}\omega }}{\left| \omega \right|} < \infty }$$

$\psi (t)$ is called base wavelet or wavelet generating function. Stretch and translate $\psi (t)$ to obtain the following function:

2 $$\psi_{a,b} \left( t \right) = \frac{1}{{\sqrt {\left| a \right|} }}\psi \left( {\frac{t - b}{a}} \right)$$

where $\psi_{a,b} (t)$ is the continuous wavelet basis function, *a* is the scale parameter, *b* is the translational parameter, and $a,\;b \in R\;(a \ne 0)$. For any function $f(t) \in L^{2} (R)$, the $\psi (t)$ is a wavelet basis function with the following continuous wavelet transform.

3 $$W_{f} \;(a,\;b) = |a|^{{ - \frac{1}{2}}} \int_{ - \infty }^{ + \infty } {f(t)\overline{{\psi \left( {\frac{t - b}{a}} \right)}} {\text{d}}t,\quad a \ne 0}$$

where, $f(t)$ is about the family of functions $\psi_{a,b} (t)$, where $\overline{{\psi \left( {\text{t}} \right)}}$ is the conjugate function of $\psi_{a,b} (t)$. The inverse of the wavelet transform is given by

4 $$f(t) = \frac{1}{{C_{\psi } }}\int_{ - \infty }^{ + \infty } {\int_{ - \infty }^{ + \infty } {a^{ - 2} W_{f} (a,\;b)\psi_{a,b} (t){\text{d}}adb} }$$

The selection of a wavelet function is a difficult point in wavelet analysis. In this paper, the wavelet functions are selected from four perspectives: branch length, vanishing distance order, regularity, and symmetry of wavelets. Finally, Daubechies wavelet is selected, and after comparing the actual filtering effect of Daubechies wavelet with different N values, it is decided to use db5 wavelet to decompose the current signal. The wavelet decomposition coefficients are obtained and presented in the form of images. The modal maxima of the wavelet coefficients reflect the sudden change point characteristics of the signal and can be used to detect arc faults. A continuous wavelet transform is performed on the original current signal with a scale of a = [1:1:64]. The wavelet transform time–frequency map is obtained. To reduce the computation of the detection model, the image features are grayed using the weighted average method. The computational principle can be expressed as follows.

5 $$Gray(i,j) = w_{R} {\text{R}} + w_{G} G{ + }w_{B} B$$

where $w_{R}$, $w_{G}$ and $w_{B}$ are the weights of R, G, and B, respectively, and different values are taken to form different grayscale images. Since the human eye is most sensitive to green, followed by red, and least sensitive to blue, making $w_{G} > w_{R} > w_{B}$ will obtain a grayscale image with easier recognition. The best grayscale image is obtained when $w_{R} = 0.299$, $w_{G} = 0.587$, $w_{B} = 0.114$.

**Figure 3:**
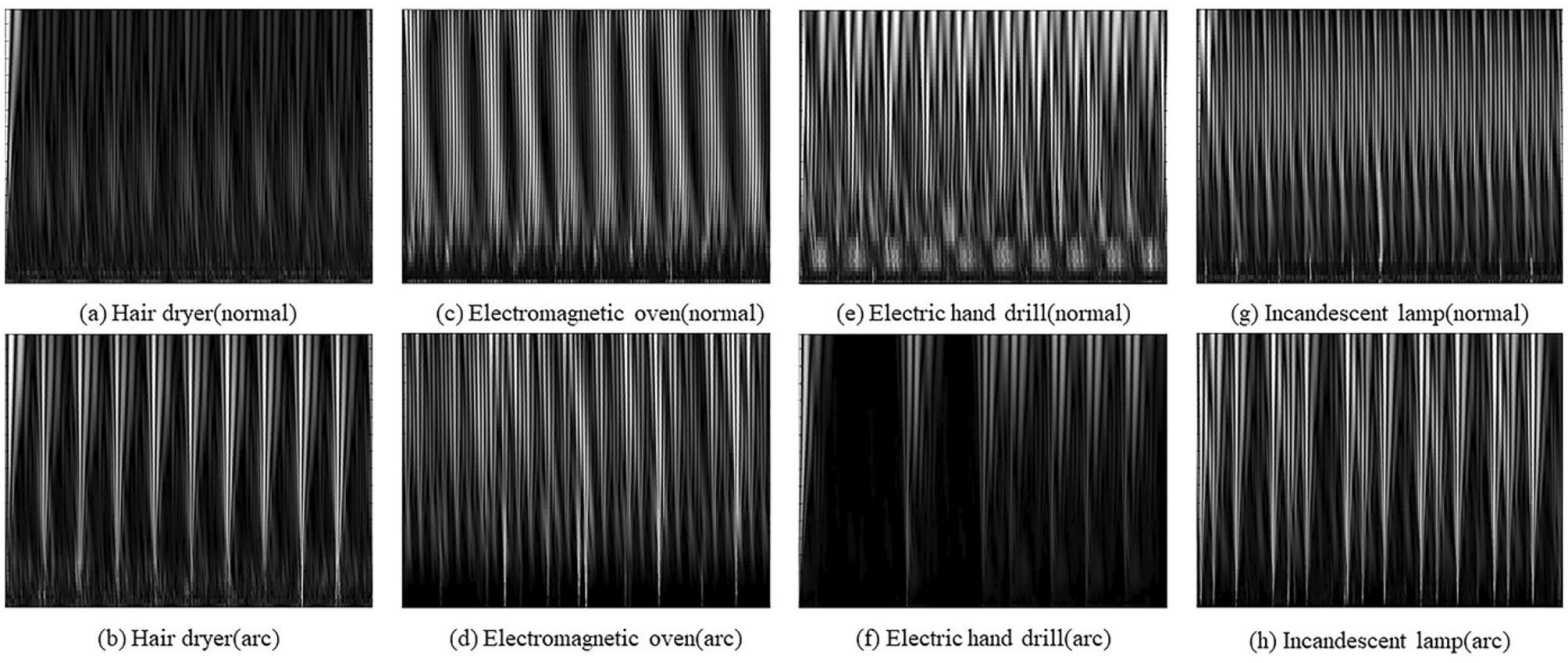
The wavelet coefficient diagram of four typical load under normal and arc fault states.

To eliminate the effect of affine transformation and accelerate the gradient descent, the original image is normalized. Finally, the processed image features are shown in Fig. 3. The calculation principle is as follows.

6 $$x(i,j) = Gray(i,j)/255$$

### Data enhancement

In the low-voltage distribution system, the occurrence of series arc faults is random and the actual arc fault data is difficult to record. Although the arc fault simulation can be realized through the experiment, the experimental process is complex and with a certain degree of danger. In addition, it is necessary to conduct experiments on the same type of loads of different sizes of common household appliances. The loads are extremely easy to be damaged during the experiments, which requires a lot of time and money investment. Therefore, the amount of data collected through experiments is generally challenging to meet the requirements of deep learning, which seriously affects the application of deep learning models in detection tasks.

Based on the imaging characteristics of the wavelet transform coefficient, four technical means are selected to augment the dataset.

1. Rotation: The image is rotated 90° clockwise with the center of the image as the original, then the dataset has been expanded by four times.
2. Mirroring: After the rotation operation, the image set is processed by the vertical mirror and horizontal mirror, and the dataset is expanded by two times.
3. Noise: The random addition of Gaussian noise with zero mean characteristics to some pictures can effectively distort the high-frequency features, weaken its impact on the model and improve the learning ability of the neural networks.
4. Displacement: The maximum amplitude of random translation is 0.2. The filling method adopts edge filling.

### DRSN-CW model

A typical convolutional neural network (CNN) generally consists of an input layer, an output layer, and multiple hidden layers. The hidden layers can be divided into convolutional, activation, pooling, and fully connected layers. The input layer is used to control the size and type of the input data. The convolutional layer is the core of the CNN, which is used to extract features. The output of the convolutional layer for an *l*-th layer can be expressed as follows.

7 $$x_{j}^{l} = f\left( {\sum\limits_{i \in M} {k_{i}^{l} {*}x_{ij}^{l - 1} + b_{j}^{i} } } \right)$$

where, $x_{j}^{l}$ is the *j*-th feature map of the *l*-th layer, *j* is the number of convolution kernels, *k* is the convolution kernel, * is the convolution operator, *M* is the number of channels of the input feature maps, $x_{ij}^{l - 1}$ is the input feature map of the *l*-th layer, $b_{j}^{i}$ is the corresponding bias vector, and *f* is the activation function used.

The activation layer is commonly used after the convolutional layer to perform a non-linear mapping of the features that are extracted from the convolutional layer. The commonly used activation function is ReLU, which can be expressed as follows.

8 $${\text{Re}} LU(x) = \left\{ {\begin{array}{*{20}l} {x,\begin{array}{*{20}l} {} \\ \end{array}\quad x > 0} \\ {0,\begin{array}{*{20}l} {} \\ \end{array}\quad x \le 0} \\ \end{array} } \right.$$

The pooling layer is mainly used to reduce the dimensionality of the feature map and extract the main features to avoid overfitting. The most popular pooling operations are MaxPooling and AveragePooling.

1. MaxPooling
  9 $$P_{i}^{{l{ + }1}} (t) = \max_{(j - 1)W + 1 \le t \le jW} \left\{ {f_{i}^{l} (t)} \right\}$$
2. AveragePooling
  10 $$P_{i}^{{l{ + }1}} (t) = \frac{1}{W}\sum\nolimits_{(j - 1)W + 1}^{jW} {\begin{array}{*{20}c} {f_{i}^{l} (t)} \\ \end{array} }$$

where, $P_{i}^{l + 1} (t)$ denotes the output value of the *t*-th neuron in the *i*-th feature map of layer *l* + 1, *W* is the area of pooling, and $f_{i}^{l} (t)$ denotes the output value of the *t*-th neuron in the *i*-th feature map of layer *l*.

The fully connected layer is used mainly to refit the features and reduce the loss of feature information. The expression of the function can be expressed as follows.

11 $$y_{i}^{{l{ + }1}} = f(w^{l} \cdot x^{l} + b^{l} )$$

where, *w* denotes the weight and *b* denotes the bias. The fully connected layer and the Softmax function, can form the output layer. For an array *X* with n elements, the Softmax value of the *i*-th element of the array can be expressed as follows.

12 $$Soft\max (x_{i} ) = \frac{{e^{{x_{i} }} }}{{\sum\nolimits_{n} {e^{{x_{n} }} } }}$$

The accuracy of the deep learning model is generally improved as the number of network layers increases. However, the training accuracy and testing accuracy drop rapidly after the network layers increase to a certain number. It shows that the problem of gradient disappearance and explosion becomes apparent, and the network even degenerates with the increase of network depth. Therefore, the residual network structure is introduced to achieve high accuracy through cross-layer connection and increasing network depth.

**Figure 4:**
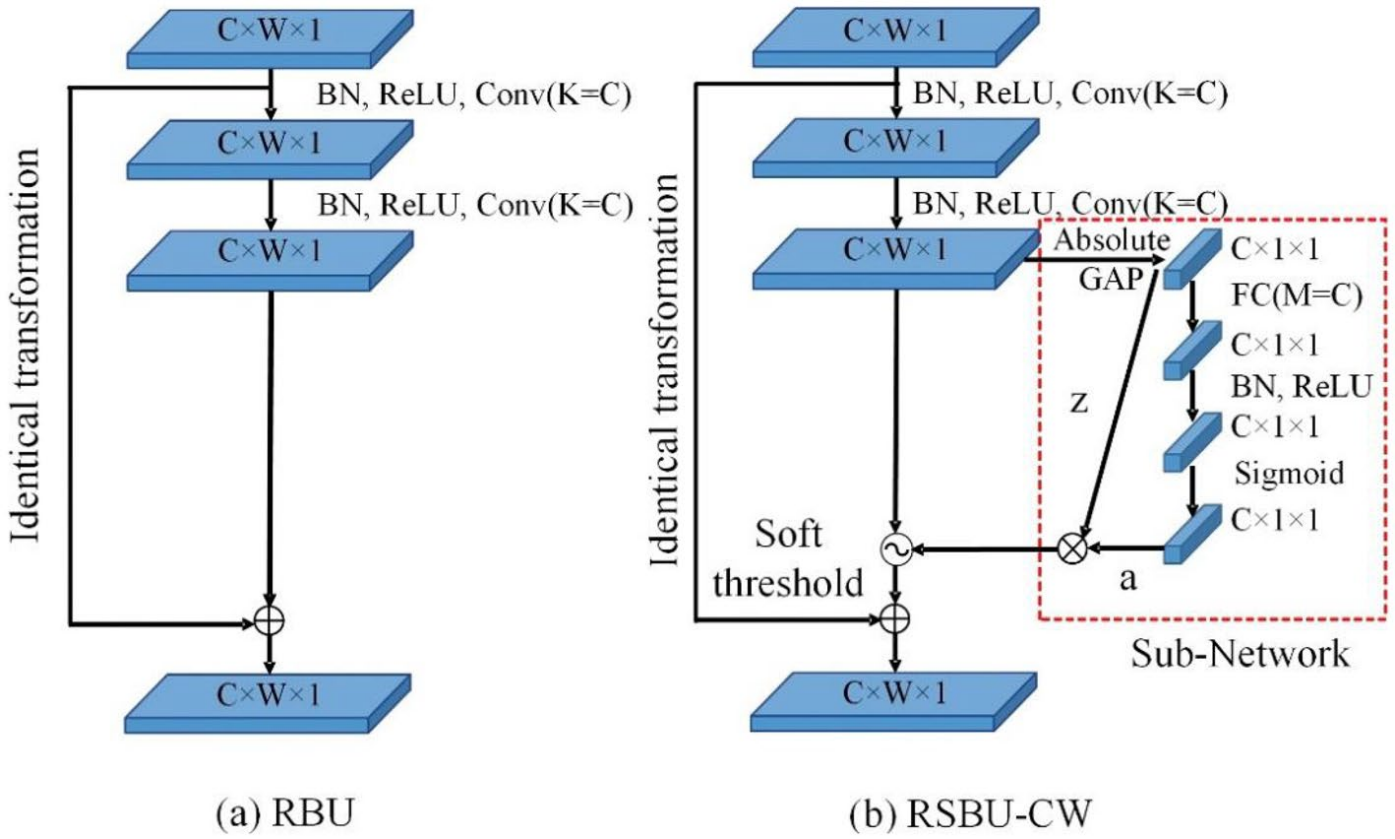
Schematic diagram of the network module structure. (**a**) Residual Building Unit (RBU). (**b**) Residual Shrinkage Building Unit with Channel-Wise thresholds (RSBU-CW).

Deep residual shrinkage network (DRSN) is a variant of deep residual network^21^. It achieves automatic soft thresholding inside the model by embedding sub-networks in the residual module. It can adaptively remove noise and redundant information in the feature learning to improve the feature learning effect. According to whether the thresholds are shared between channels, there are Residual Shrinkage Building Unit with Channel-Wise thresholds (RSBU-CW) and Residual Shrinkage Building Unit with Channel-Shared thresholds (RSBU-CS). Compared to RSBU-CS, which has only one set of thresholds, RSBU-CW is more accurate and flexible. Therefore, the DRSN-CW model is constructed by RSBU-CW in this paper. The network structure of the residual module and the residual shrinkage module is shown in Fig. 4.

Where, C denotes the number of channels of the feature mapping, W denotes the width of the feature mapping, and 1 denotes the depth of the feature mapping. K is the number of convolutional kernels in each convolutional layer, and M is the number of neurons in the fully connected layer. Batch Normalization (BN) is used to normalize the data distribution to avoid parameters falling into the saturation zone. The activation function is the ReLU function. The sub-network is a small fully connected network. The convolved feature mapping values are performed GlobalAverage Pooling (GAP) and their absolute value *z* is taken as the input to the sub-network. The Sigmoid function is used as the output activation function and the output value is scaled to between (0, 1). Finally, the input value *z* of the sub-network is multiplied with the output value *a* to obtain the value as the threshold value, which is input into the soft threshold function for eliminating noise and redundant information. The expression of the process is as follows.

13 $$z = \left| {\overline{X} } \right|$$

14 $$a_{c} = \frac{1}{{1 + e^{{ - z_{c} }} }}$$

15 $$\tau_{c} = z \cdot a_{c}$$

where, *X* is the output feature map of the second convolutional layer. $z_{c}$ is the feature of the *c*-th layer neuron, and $a_{{\text{c}}}$ is the scaling parameter of the *c*-th layer. $\tau_{{\text{c}}}$ is the threshold value of the *c*-th channel of the feature map. The expression of the soft thresholding function is given as

16 $$y = \left( {\begin{array}{*{20}l} {x - \tau}, &\quad {x > \tau } \\ 0 , &\quad { - \tau \le x \le \tau } \\ x + \tau, &\quad {x < \tau } \\ \end{array}} \right.$$

where, *x* denotes the feature input feature and *y* denotes the output feature. $\tau$ denotes the threshold value, which is a positive parameter. The derivatives are as follows.

17 $$\frac{\partial y}{{\partial x}} = \left\{ {\begin{array}{*{20}l} 1, & \quad x > \tau \\ 0, &\quad - \tau \le x \le \tau \\ 1, &\quad x < \tau \\ \end{array}}\right.$$

As above, the soft thresholding function can set the features to 0 for any interval and retain the effective negative features for better feature mapping.

## Results

### Series arc fault data set

In this paper, four typical loads were used for series arc simulation experiments. For each load, 34 sets of experiments were performed under fault and normal operating conditions and 68 sets of experimental data were obtained. In total, 272 sets of experimental data were obtained for all loads. For the 272 sets of experimental data, 272 wavelet coefficient diagrams were obtained by wavelet continuous transformation. After grayscale and data enhancement, the 272 wavelet coefficient maps were expanded to 4080, which are 510 for each operating condition of each load. The feature data after data processing were given labels to construct the dataset. The composition of the dataset is shown in Table 1.

**Table 1:** Typical loads and corresponding labels.

Load	Type	State	Source samples	Enhanced samples	Label
Hair dryer	DC motor	Arc	34	510	1
		Normal	34	510	2
Electromagnetic oven	Eddy current	Arc	34	510	3
		Normal	34	510	4
Electric hand drill	Single-phase series motors	Arc	34	510	5
		Normal	34	510	6
Incandescent lamp	Resistive leakage	Arc	34	510	7
		Normal	34	510	8

### Detection model

Because the source data samples are small, some noise and redundant data are generated while data enhancement is performed on the dataset. How to remove the noise and redundant data becomes an important issue to improve the model performance. The degree residual shrinkage network can adaptively determine soft thresholds to eliminate noise, thus solving the above issue.

This paper improves the DRSN-CW model to make the depth structure more suitable for learning the features. After repeated parameter adjustment and experiments, the improved DRSN-CW model structure mainly consists of an input layer, a convolutional layer, a residual shrinkage layer, a maximum pooling layer, a global average pooling layer, a fully connected layer and an output layer. The specific structure is shown in Table 2. The regularization is added to the convolution layers and total connection layers with the parameter *L2* = *le−4*. Dropout is added after the two fully connected layers, which is taken as 0.5. Adam is chosen as the optimizer. It can dynamically adjust the learning rate of each parameter by using the first-order and second-order moment estimation of the gradient. After bias correction, the learning rate for each iteration has a specific range to make the parameters more stable. The softmax function obtains the fault detection result.

**Table 2:** The configuration parameters of DRSN-CW model.

Number	Layer type	Convolution kernel	Number	Activation function	Dropout	Output size
1	Input	–	–	–	–	180 × 180 × 1
2	Convolution layer	3 × 3	32	Relu	–	180 × 180 × 32
3	Residual shrinkage layer	–	–	–	–	90 × 90 × 32
4	Maximum pooling layer	2 × 2	32	–	–	45 × 45 × 32
5	Convolution layer	3 × 3	64	Relu	–	45 × 45 × 64
6	Maximum pooling layer	2 × 2	64	–	–	22 × 22 × 64
7	Convolution layer	3 × 3	64	Relu	–	22 × 22 × 64
8	Maximum pooling layer	2 × 2	64	–	–	11 × 11 × 64
9	Convolution layer	3 × 3	128	Relu	–	11 × 11 × 128
10	Maximum pooling layer	2 × 2	128	–	–	11 × 11 × 128
11	Global average pooling	–	–	–	–	128
12	Fully connected layer	–	–	Relu	0.5	2048
13	Fully connected layer	–	–	Relu	0.5	1024
14	Output	–	–	Softmax	–	8

### Analysis of results

**Figure 5:**
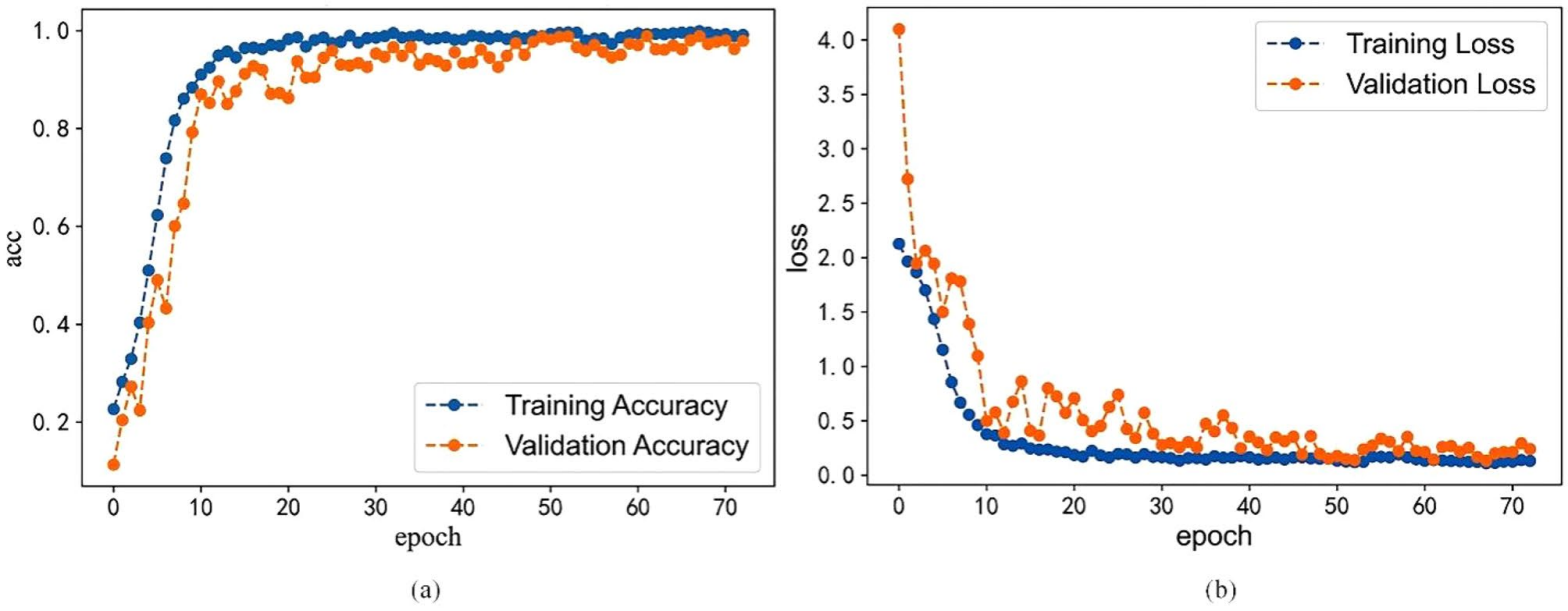
The results of the detection. (**a**) The accuracy of training and validation set. (**b**) The loss of training and validation set.

The processed data samples are divided into training sets and validation set in the ratio of 7:3. Each Batch contains 48 samples, and the callback function is used to control the training model. The monitor point is the accuracy of the validation and the duration step is 10. After 74 epochs, the accuracy of the training set reaches over 99% and tends to be stable. The training loss drops from 2.13 to 0.13 and tends to be 0. The accuracy of the validation set shows a slight oscillation. It reaches a maximum of 98.92% in the 64th epoch, and the loss drops from 4.10 to less than 0.4. The detection results are shown in Fig. 5. During the detection process, there was no overfitting, so the detection results were good.

### Results visualization

**Figure 6:**
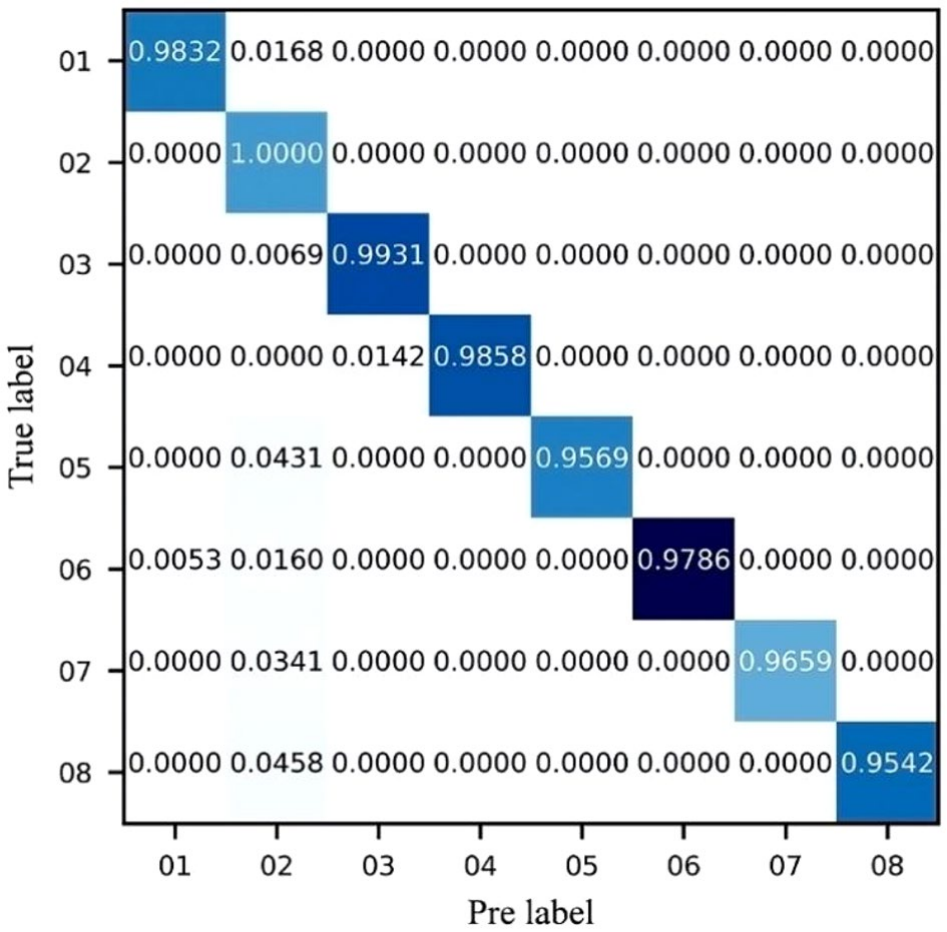
The visualization of detection result.

A confusion matrix is a visualization tool in artificial intelligence, especially for supervised learning, to compare classification results with actual measured values. The confusion matrix shows which part of the model will be confused when making predictions, and this decomposition of the results overcomes the limitations of using classification accuracy alone. DRSN-CW model detection result is visualized in the confusion matrix shown in Fig. 6.

The average accuracy is 97.72%, as shown in Table 3. The accuracy of hairdryer load in the normal state is the highest and reaches 100%. The accuracy of incandescent lamp load in normal state and the electric hand drill load in arc fault state is low, respectively 95.42% and 95.69%.

**Table 3:** Typical load identification results.

Load	State	Label	Accuracy (%)
Hair dryer	Arc	1	98.32
	Normal	2	100
Magnetic hotplate	Arc	3	99.31
	Normal	4	98.58
Electric hand drill	Arc	5	95.69
	Normal	6	97.89
Incandescent lamp	Arc	7	96.59
	Normal	8	95.42

## Discussion

To further verify the superiority of the constructed models, in this paper, five typical deep learning detection algorithms are explored under the same dataset and experimental conditions. The CNN is a feed-forward neural network with excellent performance in processing large images. AlexNet deepens the structure of the network based on CNN, which is able to learn richer and higher-dimensional image features. VGGNet inherits the idea of AlexNet. Based on AlexNet, a network is built with more layers and deeper depth is built. VGGNet mainly explores the relationship between the depth and performance of convolutional neural networks. It has good performance on different image datasets. The Inception-V3 is the third version of the Inception series of models. Inception increases the complexity of the network by increasing the network width. It can reduce the number of parameters and extract high-dimensional features while ensuring the quality of the model, and it has achieved excellent results in image classification problems. ResNet provides a residual network structure design to avoid the problem of gradient disappearance and explosion as the network depth increases. ResNet has a higher accuracy in image classification problems. DRSN, as a variant of ResNet, was first used for the detection of the fault vibration signals. Due to the introduction of soft thresholding based on the residual module, it has better results in classifying feature samples containing noisy data.

The detection models based on CNN, AlexNet, VGG-16, Inception-V3, and ResNet are constructed, respectively, and compared with the proposed DRSN- CW model. The detection accuracy of the arc fault for each model is shown in Table 4. The CNN, AlexNet, VGG-16, Inception-V3, and ResNet models produce drastic oscillations and local overfitting during training. In contrast, the proposed DRSN-CW model forces the noisy feature to 0 by a soft threshold and presents a more stable accuracy on the test set. Compared with other deep learning models, the DRSN-CW model has the highest accuracy for series arc fault detection.

**Table 4:** Each model series arc fault identification correct rate.

	Model	Maximum accuracy (%)	Average accuracy (%)
1	CNN^17^	91.09	81.60
2	AlexNet^19^	94.03	83.17
3	VGG-16^22^	96.87	94.71
4	Inception-V3^23^	97.84	88.65
5	ResNet^20^	93.84	89.53
6	DRSN-CW	98.92	97.72

## Conclusion

This paper proposes a series arc fault detection method based on continuous wavelet transform and deep residual shrinkage network with the channel-wise threshold (DRSN-CW). Furthermore, we get the following conclusions.

1. The continuous wavelet transform is used to extract the features of the current signal. The db5 wavelet is selected to decompose the current signal and obtain the wavelet coefficients after comparing the filtering effect of each wavelet function. The modal maxima of wavelet coefficients reflect the feature of sudden change points of the signal.
2. A data set enhancement method based on adding noise, rotation and mirror-image can solve the problem of fewer data.
3. The model based on DRSN-CW is constructed, which uses soft thresholds to remove noise and redundant data to improve the accuracy of detection results.
4. The detection models based on CNN, AlexNet, VGG-16, Inception-V3, and ResNet are constructed, respectively, and compared with the proposed DRSN- CW model. The DRSN-CW model has the highest accuracy for series arc fault detection with limited source data samples, which provides a new idea for arc fault detection.

## Data Availability

The datasets used during the current study are available from the corresponding author on request.
